# In-depth characterisation of the urine metabolome in cats with and without urinary tract diseases

**DOI:** 10.1007/s11306-022-01877-9

**Published:** 2022-03-17

**Authors:** Younjung Kim, Wei Xu, Vanessa Barrs, Julia Beatty, Ákos Kenéz

**Affiliations:** 1 Centre for Applied One Health Research and Policy Advice, Jockey Club College of Veterinary Medicine and Life Sciences, City University of Hong Kong, Hong Kong, SAR China; 2 Department of Infectious Diseases and Public Health, Jockey Club College of Veterinary Medicine and Life Sciences, City University of Hong Kong, Hong Kong, SAR China; 3 Beijing Research Center of Intelligent Equipment for Agriculture, Beijing, 100097 China; 4 Centre for Animal Health and Welfare, Jockey Club College of Veterinary Medicine and Life Sciences, City University of Hong Kong, Hong Kong, SAR China; 5 Department of Veterinary Clinical Sciences, Jockey Club College of Veterinary Medicine and Life Sciences, City University of Hong Kong, Hong Kong, SAR China; 6 Present Address: School of Life Sciences, University of Sussex, Brighton, BN1 9QG UK

**Keywords:** Metabolomics, Feline urine metabolome, LC–MS, Essential amino acids, Chronic kidney disease, Feline idiopathic cystitis

## Abstract

**Introduction:**

Our understanding of the urine metabolome and its association with urinary tract disease is limited in cats.

**Objectives:**

We conducted a case–control study to characterise the feline urine metabolome, investigate its association with chronic kidney disease (CKD) and feline idiopathic cystitis (FIC), and assess its compositional relationship with the urine microbiome.

**Methods:**

The urine metabolome of 45 owned cats, including 23 controls, 16 CKD, and 6 FIC cases, was characterised by an untargeted metabolomics approach using high-performance chemical isotope labelling liquid chromatography–mass spectrometry.

**Results:**

We detected 9411 unique compounds in the urine of controls and cases and identified 1037 metabolites with high confidence. Amino acids, peptides, and analogues dominated these metabolites (32.2%), followed by carbonyl compounds (7.1%) and carbohydrates (6.5%). Seven controls from one household showed a significant level of metabolome clustering, with a distinct separation from controls from other households (p value < 0.001). Owner surveys revealed that this cluster of cats was fed dry food only, whereas all but one other control had wet food in their diet. Accordingly, the diet type was significantly associated with the urine metabolome composition in our multivariate model (p value = 0.001). Metabolites significantly altered in this cluster included taurine, an essential amino acid in cats. Urine metabolome profiles were not significantly different in CKD and FIC cases compared with controls, and no significant compositional relationship was detected between the urine metabolome and microbiome.

**Conclusion:**

Our study reveals in-depth diversity of the feline urine metabolome composition, and suggests that it can vary considerably depending on environmental factors.

**Supplementary Information:**

The online version contains supplementary material available at 10.1007/s11306-022-01877-9.

## Introduction

Metabolomics is a systematic analytical approach that identifies and quantifies all metabolites and their derivatives in a biological system, such as in cells, tissues, organisms, or biological fluids. Capturing the complete set of metabolites in a biological system, i.e. the metabolome, and analysing the metabolome of individuals under different conditions provides an opportunity to understand physiological and pathological alterations of the metabolic phenotype (Weckwerth & Morgenthal, 2005).

As a biological fluid containing metabolic by-products excreted from the bloodstream, urine has been commonly analysed for metabolomics research to understand disease pathogenesis and progression and thereby to improve diagnostic and therapeutic approaches (Bouatra et al., 2013). An extensive body of research has identified around 3100 metabolites in human urine (Bouatra et al., 2013), and revealed associations between the urine metabolome and various diseases (Alonso et al., 2016; Posada-Ayala et al., 2014; Wittmann et al., 2014) and environmental conditions (Jain et al., 2019; Lindqvist et al., 2020). However, in cats, we are aware of only two studies that have characterised the urine metabolome (Broughton-Neiswanger et al., 2020; Rivera-Vélez & Villarino, 2018). While these reports have contributed to improving our knowledge of the feline urine metabolome, the set of the identified metabolites was relatively limited (i.e. 125 and 114 compounds, respectively) (Broughton-Neiswanger et al., 2020; Rivera-Vélez & Villarino, 2018), compared with the human urine metabolome (Bouatra et al., 2013), leaving the diversity of the feline urinary metabolome largely unexplored. In addition, these studies used only young, female, and intact cats from a commercial breeding company that were kept under standardised housing conditions. While such a study population enabled the analysis of the feline urine metabolome in a relatively controlled setting, the relevance of the results to owned cats where demographic (e.g. age, sex, breed) and environmental (e.g. diet and cohabitation) factors vary, potentially affecting urine metabolome composition, is unknown.

Furthermore, no studies have yet assessed the association between the urine metabolome and urinary tract diseases in cats. Chronic kidney disease (CKD) and feline idiopathic cystitis (FIC) are common causes of morbidity and mortality in cats. Not only is urine the route of excretion of many metabolic by-products, but urine metabolome composition could also be affected by kidney and bladder diseases locally altering the metabolism of these tissues (Posada-Ayala et al., 2014; Wittmann et al., 2014). Therefore, we hypothesised that cats with CKD and FIC have distinct metabolic patterns compared with cats that are free from these diseases. Assessing this hypothesis could provide novel insight into the associated biochemical processes, and possibly reveal new disease biomarkers. Finally, despite associations between the metabolome and microbiome observed in other body compartments (McHardy et al., 2013; Stewart et al., 2017), their association in the urine has not been investigated.

Here, we conducted a case–control study among cats attending first-opinion veterinary clinics in Hong Kong to (i) characterise the urine metabolome in control cats free from urinary tract diseases, (ii) assess associations of demographic and environmental factors with the urine metabolome, (iii) compare the urine metabolome between control cats and cats diagnosed with stage 2 CKD (CKD2) or FIC, and (iv) assess a compositional relationship between the urine metabolome and microbiome.

## Methods

### Study design

This study was designed as a case–control study targeting cats attending two first-opinion veterinary hospitals in Hong Kong. The study used residual urine samples collected for diagnostic purposes with informed and written owner consent, and was exempted from animal ethics approval by the Animal Research Ethics Sub-Committee, City University of Hong Kong. Demographic and clinical information were obtained from electronic patient records with owner consent, and dietary information was obtained by owner survey for each cat. Case and control definitions and their selection criteria are summarised in Table 1.

**Table 1:** Case and control definitions and selection criteria

Case and control definitions		
Stage 2 Chronic Kidney Disease (CKD) • Urine specific gravity (USG) less than 1.035 • And serum creatinine concentration greater than 140 and less than 250 µmol/ml (International Renal Interest Society, 2019) • And no dietary intervention for CKD	Feline idiopathic cystitis • Presented for one or more of lower urinary tract signs (LUTS), including pollakiuria, stranguria, periuria, dysuria, or haematuria • And diagnostic investigation, including, in all cases, physical examination, urinalysis, urine culture, and abdominal ultrasonography, failed to identify a specific cause of LUTS	Control • Presented for reasons other than CKD or LUTS • And no history of LUTS in the last 3 months • And no evidence of CKD and FIC by diagnostic test results

Selection criteria		
Inclusion criterion • Both urinalysis and urine culture results available for a sample collected via ultrasound-guided cystocentesis		
Exclusion criteria • Systemic antimicrobial treatment or urinary catheter placement in the last 3 months • Or insufficient residual urine for metabolomics (< 200 µL) • Or culture-positive urine culture		

Residual urine was stored at 4 °C in the clinic, transported to City University of Hong Kong on ice, aliquoted for metabolomics (200 µL) and microbiome (1 mL) analyses, and then frozen at − 80 °C within 90 min of collection.

### Metabolomics analysis

For in-depth urine metabolomics, a high-performance chemical isotope labelling (CIL) liquid chromatography–mass spectrometry (LC–MS) was performed at the Li-Node of The Metabolomics Innovation Centre (TMIC), the Department of Chemistry, University of Alberta (Edmonton, Canada) according to the published analytical workflow (Zhao et al., 2016, 2019). All samples were processed in one batch within 5 months after sample collection. Briefly, for each sample, a proprietary metabolome quantification kit from Nova Medical Testing Inc. (Edmonton, Canada; Product Number: NMT-6001-KT) was used to measure the total metabolite concentration in an aliquot of 25 µL following the standard operating procedure of the kit. All samples were then normalised to 16 mM with LC-MC grade water based on the total metabolite concentrations and centrifuged at 12,000×*g* for 10 min (Wu & Li, 2012, 2016). The supernatant of each sample was split into 4 aliquots, diluted with LC–MS grade water to 4 mM in the total volume of 25 μL, and then subject to ^12^C or ^13^C isotope labelling. Separately, a pooled sample was prepared by mixing all ^13^C-labelled samples in equal volumes of 60 μL. The isotope labelling targeted the following four chemical-group-submetabolome channels: (i) amine/phenol, (ii) carboxyl, (iii) hydroxyl, and (iv) carbonyl. Finally, among samples labelled for the same channel, each ^12^C-labelled sample was mixed with the ^13^C-labelled pooled sample in equal volumes and analysed by LC–MS. Four quality-control samples were included in the LC–MS analysis of each channel.

From the data generated by CIL LC–MS, only peak pairs that were present in at least 80% of samples in any study groups were retained (Smilde et al., 2005; Yang et al., 2016). Based on mass and retention time (where available), peak pairs were searched against a labelled metabolite library (CIL Library), linked identity library (LI Library), and then MyCompoundID library (MCID Library). Those matched to CIL, LI, and MCID libraries were classified as Tiers 1, 2, and 3 compounds, respectively. The identification of Tier 1 (positively annotated) and Tier 2 (highly confidently annotated) compounds was considered adequate for metabolic analyses requiring metabolite identification, whereas Tier 3 classification was equivalent to unannotated metabolic features and chemical formulas. Therefore, Tiers 1, 2, and 3 reported herein corresponded to the Metabolomics Standard Initiative Levels 1, 2, and 3, respectively (Sumner et al., 2007).

### Microbiome data

DNA extraction, PCR amplification, 16S rRNA gene sequencing, and bioinformatics were performed as described in Kim et al. (2021). We assessed the sequencing depth of individual microbiome samples based on how Shannon diversity, one of the alpha diversity metrics, changed over different counts of amplicon sequence variants (ASV) on a rarefaction curve. We then identified an ASV count from which Shannon diversity began to saturate in most samples and chose samples whose ASV count was above this threshold for comparing the urine metabolome and microbiome, assuming that the sequencing depth was sufficient to represent microbial communities.

### Statistical analysis

The t tests, fold-change, pathway analysis, and enrichment analysis of the metabolomics dataset were performed in MetaboAnalyst 5.0 (Pang et al., 2021). A t test with false discovery rate (FDR) correction was conducted for each identified compound to compare its normalised peak ratios between different groups of cats. Annotated (Tiers 1 and 2) compounds were used for pathway analysis (i.e. over-representation analysis and pathway enrichment analysis) and quantitative enrichment analysis. First, we identified metabolic pathways associated with the annotated compounds through over-representation analysis, based on compound names. We then investigated metabolic pathways that were significantly altered between groups of cats with pathway enrichment and quantitative enrichment analyses, based on the normalised peak ratios of the compounds. Both pathway and quantitative analyses used the KEGG Pathway Database (www.genome.jp/kegg/pathway.html) for *Homo sapiens* as a reference pathway library because no database for *Felis catus* was available in the MetaboAnalyst interface. For pathway analysis of a given metabolic pathway, the global test was used to assess statistical significance for its enrichment, and relative betweenness was used to measure the centrality of the matched compounds on the metabolic pathway.

The remainder of the analytical workflow was performed in R.4.0.2 (R Core Team, 2020). First, we performed a multidimensional scaling (MDS) to assess the clustering of the urine metabolome between groups of cats. Briefly, we computed Bray–Curtis dissimilarity for all possible pairs of samples based on normalised peak ratios of all the identified compounds by using the *vegdist* function of the *vegan* package. Then, based on these values, we ordinated samples in reduced space through MDS by using the *cmdscale* function. We also performed the permutational multivariate analysis of variance (PERMANOVA) test by using the *adonis* function of the *vegan* package to assess statistical significance for the clustering of the urine metabolome between different groups. Second, we performed a Procrustes analysis to investigate the association between metabolome and microbiome compositions using the *procrustes* function of the *vegan* package. We also performed procrustean randomisation tests using the *protest* function of the *vegan* package to assess statistical significance for the compositional association between these two compositions. Finally, p value ≤ 0.05 was considered to indicate statistical significance.

## Results

This study was conducted on 45 cats, comprising 23 controls, 16 CKD2, and 6 FIC cases. The interquartile range of age was between 7 and 12 years. All of the cats were neutered, with males comprising 55.6% (n = 25). Around half (n = 23) were domestic short hair, and the remaining cats were pure breeds (n = 22). There was a significant age difference by disease status, with CKD2 cases being older (p value < 0.001) and FIC cases being younger than controls (p value < 0.001) (Table S1). In contrast, sex and breed were not associated with disease status. The median number of cats per household was 1, ranging between 1 and 7 (Fig. S1).

**Fig. 1:**
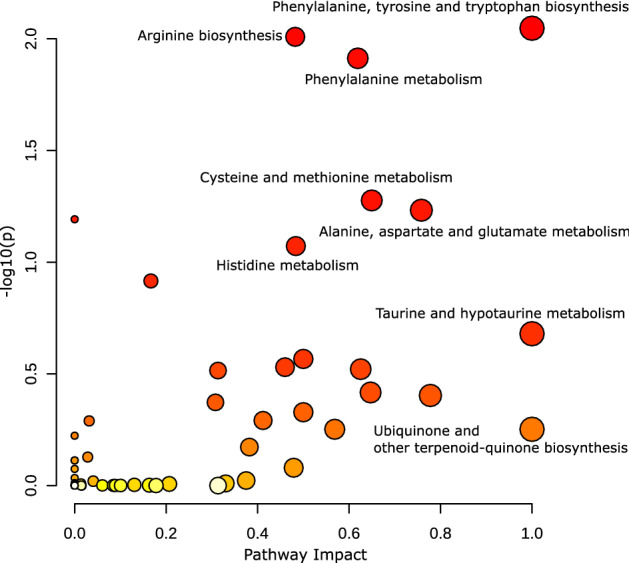
Pathway over-representation analysis of the urine metabolome of cats (n = 45). A list of Tier 1 and Tier 2 compounds was analysed by the MetaboAnalyst pathway analysis module to identify metabolic pathways associated with those compounds. The x-axis represents the pathway impact of a given metabolic pathway computed by the sum of relative-betweenness centrality of the matched metabolites, and the y-axis represents the log-transformed p-value from the hypergeometric test, with the circles with higher statistical significance expressed with more reddish colours

A total of 9,411 unique compounds were detected in the urine samples, with the number per cat ranging between 8514 and 9286 (median: 9178) (Supplementary Data). Of these, 1037 (11.0%) compounds were classified as Tier 1 or Tier 2 non-isomeric compounds (i.e. known chemical identity with positive annotation and highly confident annotation, respectively). Amino acids, peptides, and analogues (32.2%) dominated these compounds with known subclass annotation, followed by carbonyl compounds (7.1%), carbohydrates and carbohydrate conjugates (6.5%), and fatty acids and conjugates (5.6%) (Tables S2, S3). The over-representation analysis matched Tiers 1 and 2 compounds to 57 metabolic pathways from the KEGG database (Fig. 1 and Table S3). Seventeen (29.8%) of these metabolic pathways were associated with the metabolism of amino acids, including those that must be provided to cats through diet as essential amino acids: taurine, arginine, methionine, and cysteine (Fig. 1 and Table S3).

**Fig. 2:**
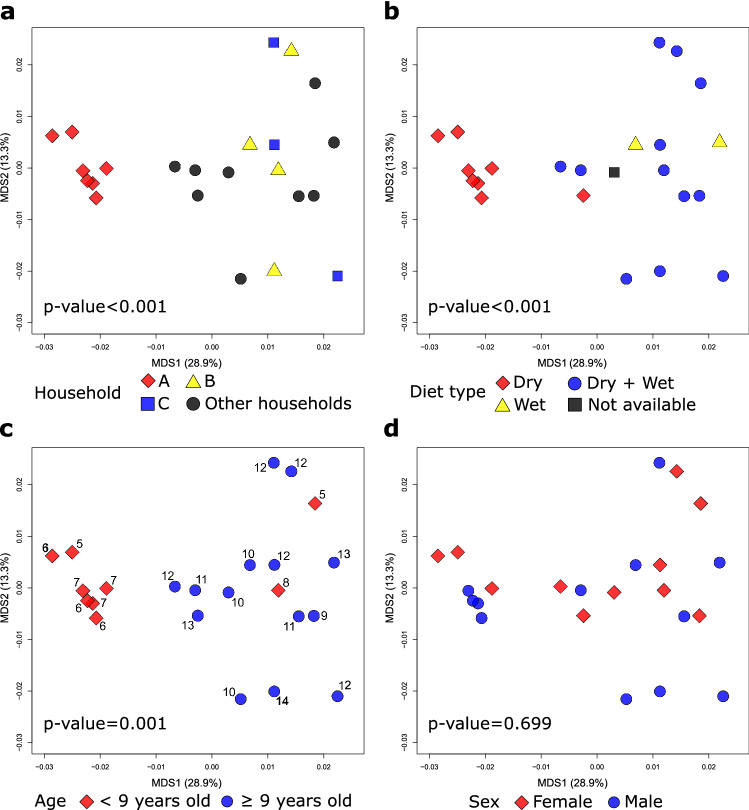
Multidimensional scaling (MDS) of the urine metabolome of control cats (n = 23). Percentages on the MDS axes represent variance explained. Symbols represent individual cats, and colours represent their household (**a**), diet type (**b**), age group (**c**) and sex (**d**). In A, cats from different households (i.e. only one cat was sampled in the given household) were labelled as “other households”. In C, numbers next to symbols represent ages in years. Cats were coloured differently using the age of 9 as the threshold given that cats are considered to enter senior years and have increasing risk of CKD at around this age (Conroy et al., 2019). The p values were obtained by the permutational multivariate analysis of variance (PERMANOVA) test. Only cats from households A, B, and C were included in the PERMANOVA test for the association between the urine metabolome composition and household since only one cat was sampled per household for cats in “other households”. Age was provided as a continuous variable in the PERMANOVA test

We investigated factors associated with the urine metabolome composition of control cats. The MDS ordination, based on all detected compounds, showed that 7 control cats from one particular household (‘household A cats’) showed a significant level of metabolome clustering, with a distinct separation from other control cats (p value < 0.001, Fig. 2a). The owner surveys revealed that these cats were fed dry food exclusively, whereas all but one other control cat were fed wet food exclusively or in combination with dry food (p value < 0.001, Fig. 2b). This cluster of cats was also younger than the rest of the study population (p value < 0.001, Fig. 2c). However, in the final model accounting for both diet type and age, while diet type remained significantly associated with urine metabolome composition (p value = 0.001), age did not (p value = 0.192). There was no evidence to support the association between urine metabolome composition and sex (Fig. 2d).

**Fig. 3:**
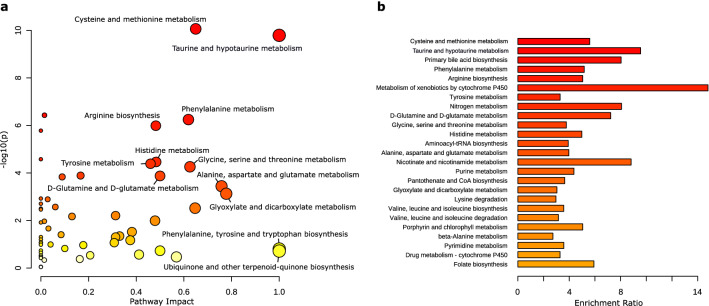
Pathway enrichment (**a**) and qualitative enrichment (**b**) analyses comparing urine metabolome of cats from household A (n = 7) and other households (n = 16). In A, the x-axis, ‘pathway impact’, represents the sum of relative betweenness centrality of the metabolites matched to each metabolic pathway. For each circle, its size is proportional to its pathway impact. In B, the x-axis, ‘enrichment ratio’, represents the observed Q statistic over the expected Q statistic. The Q statistic was obtained by averaging the squared covariance between compound concentration changes and the outcome (i.e. household A vs other households) over all compounds. In both Figures, pathways with higher statistical significance expressed with more reddish colours. The FDR-adjusted p-values are provided in Table S6

The observed clustering of household A cats away from other control cats was confirmed by both metabolite-level and pathway-level analyses. First, t tests showed that 944 (10.0%) compounds were significantly increased or decreased in household A cats. Of these, 83 were Tier 1 or Tier 2 non-isomeric compounds (Tables S4, S5). In particular, some of these compounds were identified as important metabolites for amino acid metabolism, including essential amino acids and their derivatives, such as taurine (fold change [FC]: 3.03, p value < 0.001) and 3-sulfino-l-alanine (FC: 5.07, p value < 0.001) for taurine and hypotaurine metabolism, and l-cystathionine (FC: 8.83, p value < 0.001) for methionine metabolism (Tables S4, S5). Second, significant perturbations in household A cats were identified in 28 metabolic pathways (FDR p value < 0.05) by the pathway enrichment analysis (Fig. 3a) and the quantitative enrichment analysis (Fig. 3b) (Table S6). Some metabolic pathways, for example, Metabolism of xenobiotics by cytochrome P450, had a relatively high enrichment ratio, whereas their matched metabolites had no pathway impact in terms of network topology (Table S6). Ten of those significantly perturbed pathways were associated with the metabolism of essential amino acids in cats (Table S6).

**Fig. 4:**
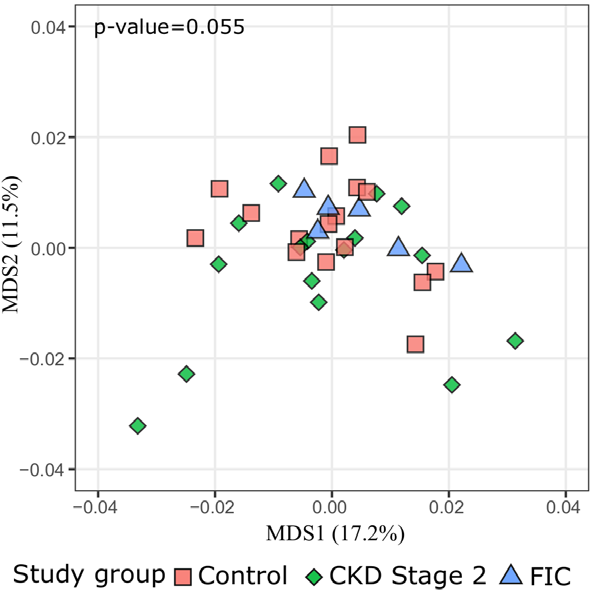
Multidimensional scaling (MDS) of the urine metabolome of control cats (n = 23), CKD (n = 16), and FIC cases (n = 6)

Including household A cats as controls could confound the association between controls and CKD2 or FIC cases, due to the significant level of their metabolome clustering. Therefore, these cats were removed from the control group for comparisons with CKD2 and FIC cases. The MDS ordination showed no clear separation of CKD2 and FIC cases from controls, and there was only weak statistical evidence for the overall difference in metabolome composition between these study groups (p value = 0.055) (Fig. 4). The pairwise comparison also revealed no evidence for a compositional difference between study groups (p value = 0.062 for CKD2 cases vs controls, 0.368 for FIC cases vs controls). In line with this observation, no compounds or metabolic pathways were found significantly altered in CKD2 and FIC cases, compared with controls (FDR p value > 0.05).

**Fig. 5:**
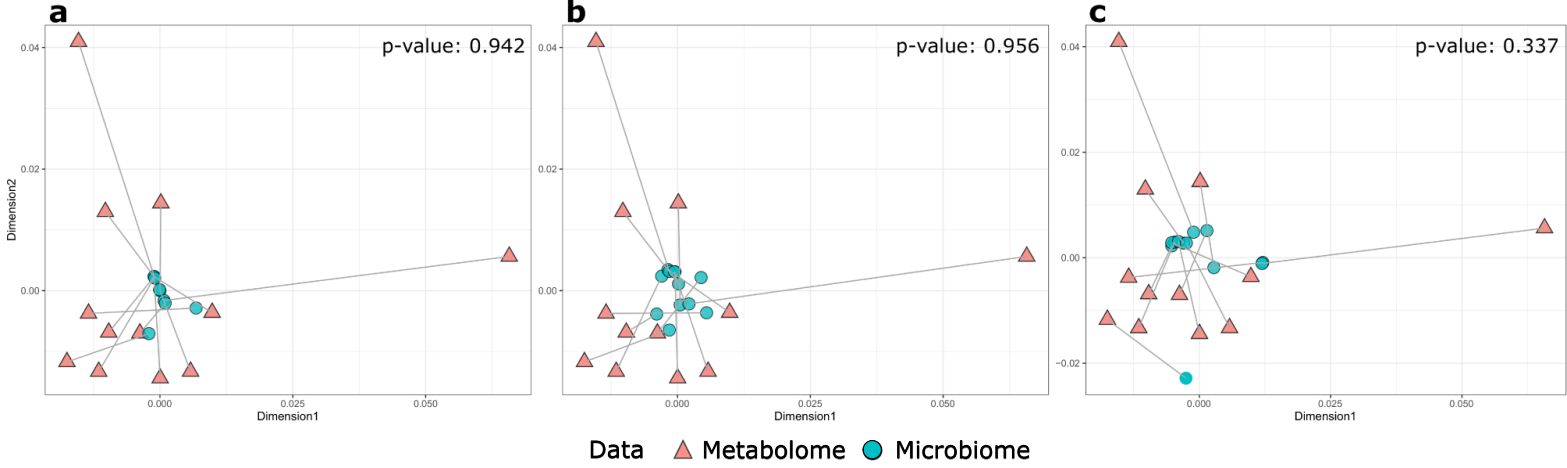
Procrustes analysis of the association between the urine metabolome and microbiome composition of cats. Only samples that passed the rarefaction curve analysis of microbiome data (i.e. > 500 16S rRNA sequences retained) were included (n = 12). The MDS ordination of metabolome data was based on Bray–Curtis dissimilarity, and the ordination of microbiome data was based on Bray–Curtis dissimilarity (**a**), unweighted Unifrac (**b**), and weighted Unifrac (**c**). One of the two ordinations were uniformly scaled and rotated until the squared differences between them were minimized, followed by the procrustean randomization test to assess the correlation between the two ordinations. Samples from the same cats are connected by a line, with orange triangles and blue circles representing samples positioned by metabolome and microbiome composition, respectively. The result of this analysis suggested no statistical evidence for the association between metabolome and microbiome composition

After the 16S rRNA gene sequencing, the sequencing depth was deemed sufficient for 12 (27.9%) cats to represent their microbial communities (Kim et al., 2021). These cats included 6 CKD2 and 4 FIC cases, as well as 2 cats with no urinary tract diseases. The Procrustes analysis revealed that there was no significant compositional association between the urinary metabolome and microbiome of these cats (Fig. 5).

## Discussion

Here, we characterised the feline urine metabolome of controls, CKD2, and FIC cases, through a global untargeted metabolomics approach. Our in-depth untargeted metabolomics assay revealed an unprecedented extent and diversity of metabolites in the urine of owned cats that were or were not affected by CKD2 and FIC. The metabolome profiles characterised herein were dominated by amino acids, peptides, and their analogues, followed by carbonyl compounds, carbohydrates, and fatty acids. Cats of the same household that were fed dry food only showed a significant level of metabolome clustering, mainly determined by amino acids, including essential amino acids in cats. However, in contrast to our initial hypothesis, urine metabolome profiles were not found significantly altered in CKD2 and FIC cases, compared with controls. The urine metabolome shared no significant compositional relationship with the urine microbiome.

We identified 1,037 metabolites with high confidence, significantly extending our previous understanding of feline urine metabolite composition. In addition, we detected 8374 other unique chemical features, demonstrating the vast diversity in the feline urine metabolome composition. As opposed to traditional LC–MS based metabolomics techniques, the methodological novelty of our study was the application of a high-performance CIL LC–MS based assay, which allowed a highly comprehensive characterisation of the metabolome with superior metabolite coverage. The unique solution that CIL LC–MS employs to extend the metabolite coverage is that it divides the metabolome into four chemical subgroups through CIL and then analyses these individual chemical-group-submetabolomes by LC–MS (Zhao et al., 2016, 2019). In addition, our study was the first to employ any LC–MS to characterise the feline urine metabolome. Different analytical techniques, particularly the ones with a relatively narrow range of metabolite coverage, are known to have a bias towards certain chemical compound classes (Weckwerth & Morgenthal, 2005). Reflecting this, the metabolome composition of the studied cats tended to be different from a previous study that applied gas chromatography/time-of-flight/mass spectrometry (GC-TOF-MS) (Rivera-Vélez & Villarino, 2018). Notably, while carbohydrates and their conjugates were dominant urine metabolites in that study, amino acids, peptides, and analogues dominated the urine metabolome in our study. More research using different analytical platforms will further increase our understanding of the feline urine metabolome, as it has been the case for the human urine metabolome (Bouatra et al., 2013).

The metabolome clustering observed in household A cats suggests that cats under the influence of the same environment likely have a more similar urine metabolome than to those from different environments. As has been the case for human urine metabolomics studies (Sampson et al., 2013; Saude et al., 2007), understanding such metabolome clustering patterns provides essential information for planning metabolomics research on spontaneously occurring diseases, particularly when using owned cats as subjects. This is because the level of metabolome clustering within groups could influence the number of samples required to detect an association, i.e. the stronger the clustering, the larger the sample size required. Furthermore, when cats from the same group are associated with a particular outcome of interest, metabolome clustering could bias the association between the urine metabolome and the outcome. Indeed, all household A cats were recruited as controls, and therefore were subsequently excluded from comparisons with CKD2 and FIC cases to avoid false conclusions. However, this post hoc approach resulted in a smaller sample size, thereby reducing statistical power of the present study.

Our findings suggest that the diet type was likely involved in the clustering of household A cats. Some of the metabolic pathways significantly perturbed in the urine of these were associated with the metabolism of essential amino acids in cats, including taurine, methionine, cysteine, arginine, and phenylalanine. Essential amino acids must be supplied through the diet because they cannot be synthesised (sufficiently) in the body. In particular, among amino acids cats require from their diet, taurine was significantly enriched in the urine of household A cats compared with the rest of the control cats. In addition, its by-product, 3-sulfino-l-alanine was also significantly enriched. This, together with the results from the pathway analysis results, suggests that the significant alterations of essential amino acids and their by-products in the urine of household A cats might have been influenced by excess bioavailability of these amino acids from the diet. In support of this, a previous study reported a significant increase in urinary taurine excretion among cats with excess dietary taurine intake (Glass et al., 1992). However, this conclusion remains speculative as we have no information on the ingredients and chemical composition of the cats’ diet beyond its type (wet or dry). For example, the difference in urinary taurine excretion was likely influenced also by other dietary factors influencing bile acid production because the conjugation of bile acids occurs almost exclusively with taurine in cats (Rabin et al., 1976). Moreover, the great diversity of commercial cat foods (e.g. variation in protein source or being grain-free) and lack of regulation as to their contents may explain the diversity in feline urine metabolome, similar to the human urine metabolome, which was shown to be affected by dietary habits either directly or through alterations of the gut microbiome (Jain et al., 2019). In contrast to the majority of the other cats, household A cats were fed dry food exclusively, which is processed with dry heating during the manufacturing process, while wet food is processed with moist heating (i.e. autoclave). The heating process was suggested to influence the nutrient bioavailability in cat food and other animal feed (Peng et al., 2014, Samadi and Yu, 2011, Hamper et al., 2016, Hickman et al., 1992, Hickman et al., 1990, de-Oliveira et al., 2012). For example, heat processing of cat food was associated with decreased digestibility of crude protein (Hamper et al., 2016), and with increased intestinal taurine loss (Hickman et al., 1990, 1992). These findings suggest that the amount of absorbed nutrients can vary depending on the type of diet and therefore influence energy metabolism in cats.

Recent metabolomics studies in humans have identified several urine metabolites as potential CKD biomarkers (Chen et al., 2019; Duranton et al., 2014; Posada-Ayala et al., 2014; Weiss & Kim, 2012; Zhao, 2013) and painful bladder syndrome/interstitial cystitis (PBS/IC) (Fukui et al., 2009; Kind et al., 2016; Parker et al., 2016; Van et al., 2003; Wen et al., 2015), the latter of which shares similarities to FIC. Our study was the first to employ a metabolomics approach to investigate the association of the feline urine metabolome with CKD and FIC. Among the four different CKD stages (International Renal Interest Society, 2019), we focused on stage 2 (CKD2) to detect metabolic patterns differentiating the early CKD stages. Urine is created through complex filtration, reabsorption, and secretion processes in the kidneys, and excreted through the lower urinary tract. Therefore, we hypothesised that the urine metabolome is altered in cats with these urinary tract diseases. However, we identified no significant alterations of urine metabolome profiles of cats with CKD2 and FIC, compared with control cats. Beyond biological reasons, the unexpected lack of associations could also be explained by potential study limitations. First, considering that FIC diagnosis is made by exclusion, FIC has a broad disease spectrum with diverse pathogenic mechanisms. Therefore, FIC cases’ urine metabolome could still be heterogeneous, hampering the detection of associations with a small sample size. Another factor that may have hampered associations being identified between disease phenotype and urine metabolome is potential confounding by age. Considering that age has been associated with the urine metabolome (Rist et al., 2017) and an independent risk factor for CKD (Sparkes et al., 2016), the age difference could have confounded the association of the urine metabolome with CKD and FIC. However, despite our intention, the availability of residual urine in our clinical settings did not allow us to better match the cats of the diseased and control groups by age. In particular, young cats seldom required urinalysis and urine culture for reasons other than LUTS. Also, old cats requiring urinalysis and urine culture generally had other accompanying diseases and were excluded from the control group. Recent studies that investigated the association of the urine metabolome with chronic hepatic diseases (Lawrence et al., 2019) and CKD (Ferlizza et al., 2020) in owned dogs recruited from veterinary hospitals also had similar issues with regard to controlling for potential confounders, such as age, sex, and diet. Although matching or focusing on specific strata could be used to account for potential confounding, such attempts likely make recruiting a sufficient number of study participants challenging in case–control studies targeting owned pets with stringent selection criteria. Therefore, future research should minimise selection bias and account for potential confounders while satisfying the practicality of urine sample collection in clinical settings.

Our study was also the first to explore the association between the feline urine metabolome and microbiome. The Procrustes analysis was unable to detect any statistically significant association between the urine metabolome and microbiome. This contrasts to a compositional similarity observed between the gut metabolome and microbiome in humans (McHardy et al., 2013). Non-significant results in this aspect are not surprising because the urinary tract microbiome is typically sparse, as shown in our previous study (Kim et al., 2021). In particular, none of the samples tested positive under routine culture, suggesting that the contribution of microbial metabolites to the overall metabolome composition was not significant in the present study. However, the Procrustes analysis likely had a limited statistical power due to the relatively small sample size. In particular, the feline urine microbiome was typically sparse and could therefore be highly variable influenced by host and environmental factors or sample collection and processing steps. These considerations suggest that future studies might require a larger sample size and controlling for potential confounding to detect hypothesised associations (Kim et al., 2021).

In conclusion, our study reveals the diverse composition of the feline urine metabolome. A significant level of metabolome clustering in a group of cats suggests that the feline urine metabolome is influenced by environmental factors, potentially by diet, which should be considered when selecting study participants in future metabolomics research on spontaneously occurring diseases in owned cats.

## Supplementary Information

Below is the link to the electronic supplementary material.

Supplementary file1 Fig. S1 Distribution of the number of cats per household (PNG 18 kb)

Supplementary file2 Supplementary Data.xlsx List of peak pairs detected from CIL LC-MS (XLSX 5442 kb)

Supplementary file3 Supplementary Tables.xlsx (XLSX 77 kb)
