# Supplementary figures and images for: In-depth characterisation of the urine metabolome in cats with and without urinary tract diseases
Source: Metabolomics. 2022 Mar 17;18(4):19. doi: 10.1007/s11306-022-01877-9 (PMC8934335; doi:10.1007/s11306-022-01877-9)

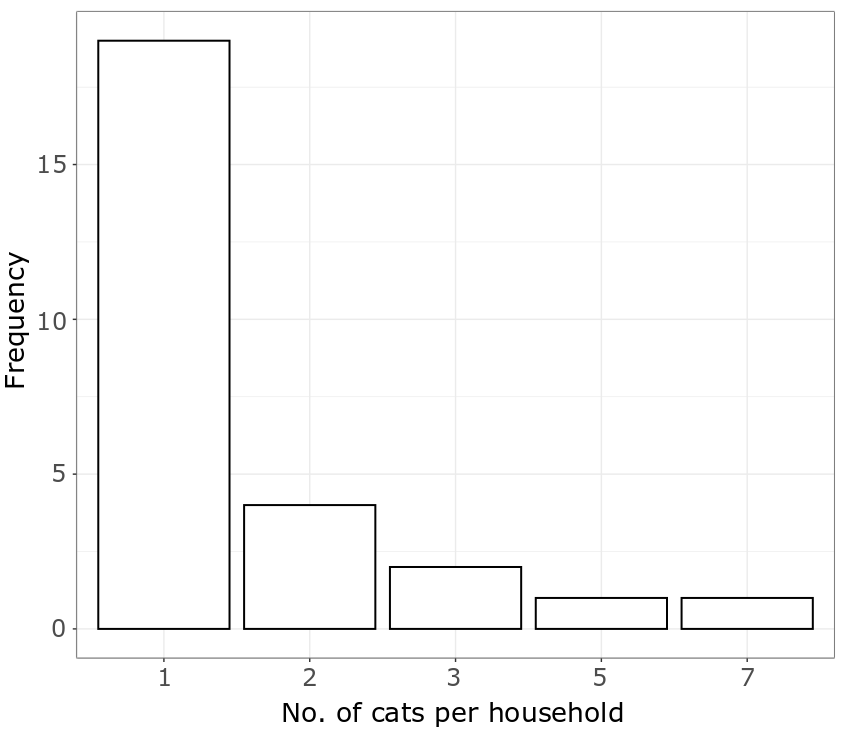

Supplement: Supplementary file 1 — Supplementary file1 Fig. S1 Distribution of the number of cats per household (PNG 18 kb) [file 11306_2022_1877_MOESM1_ESM.png]
